# *Cola rostrata* K. Schum. constituents induce cytotoxicity through reactive oxygen species generation and mitochondrial membrane depolarisation

**DOI:** 10.37349/etat.2023.00200

**Published:** 2023-12-28

**Authors:** Babatunde E. Ajayi, Bola Oboh, Joseph B. Minari, Darren W. Sexton, Satyajit D. Sarker, Amos A. Fatokun

**Affiliations:** NGO Praeventio, Estonia; ^1^Department of Cell Biology and Genetics, Faculty of Science, University of Lagos, Akoka, Lagos, Nigeria; ^2^Department of Biochemistry, Faculty of Natural and Applied Sciences, Hallmark University, Ijebu-Itele, Ogun State, Nigeria; ^3^Centre for Natural Products Discovery (CNPD), School of Pharmacy and Biomolecular Sciences, Faculty of Science, Liverpool John Moores University, L3 3AF Liverpool, UK; ^4^School of Pharmacy and Biomolecular Sciences, Faculty of Science, Liverpool John Moores University, L3 3AF Liverpool, UK

**Keywords:** *Cola rostrata*, bioassay-guided fractionation, cytotoxicity, reactive oxygen species, mitochondrial membrane potential

## Abstract

**Aim:**

While the traditional use of *Cola rostrata* in treating illnesses and diseases has not been reported, the presence of cytotoxic principles has been reported in phylogenetically and biogeographically related species within the Cola genus. This study, therefore, evaluated the cytotoxic potential of extracts of the plant, and the associated cellular and molecular mechanisms.

**Methods:**

Activity-based fractionation of the extracts was carried out and cytotoxicity was assessed in the human cervical cancer cell line, HeLa, and the transformed human lung cell line, MRC5-SV2, using the 3-(4,5-dimethylthiazol-2-yl)-2,5-diphenyltetrazolium bromide (MTT) assay complemented with brightfield imaging. The 2ʼ,7ʼ-dichlorofluorescein diacetate (DCFDA) assay was used to assess induction of cellular reactive oxygen species (ROS), while flow cytometry of 5,5ʼ,6,6ʼ-tetrachloro-1,1ʼ,3,3ʼ-tetraethyl-imidacarbocyanine iodide (JC-1)-stained cells assessed the loss of mitochondrial membrane potential (∆Ψ_M_). Gas chromatography-mass spectrometry (GC-MS) analysis was carried out on an active fraction.

**Results:**

Extracts of the fruit epicarp and leaf were cytotoxic against the cell lines. Half-maximal inhibitory concentration (IC_50_) values for the 48 h cytotoxicity of the ethanol extract of the epicarp against HeLa and MRC5-SV2 cells were 48.0 μg/mL ± 12.1 μg/mL and 40.4 μg/mL ± 7.2 μg/mL, respectively, while fractions from second-level partitioning of the hexane fraction of the leaf extract elicited cytotoxicity with IC_50_ values ranging from 12.8 μg/mL ± 1.0 μg/mL to 39.6 μg/mL ± 7.2 μg/mL in both cell lines, following 48 h treatment. GC-MS revealed the presence of seventeen compounds in a hexane fraction of the leaf extract, including even- and odd-chain fatty acids, the most abundant of which were *n*-hexadecanoic acid, decanoic acid 10-(2-hexylcyclopropyl); and octadecanoic acid. The mechanisms of cytotoxicity of most active fractions involved generation of ROS and mitochondrial membrane depolarisation.

**Conclusions:**

The findings show that *C. rostrata* is rich in cytotoxic phytochemicals which could be isolated for developing new anti-cancer agents.

## Introduction

Cancer is a disease characterized by uncontrolled proliferation of cells, deranged cellular metabolism, progressive genetic instability, de-differentiation of cells, and metastasis, which, if not detected early enough and appropriately treated, results in death [1, 2]. Currently, cancer is a leading cause of death globally, owing, in part, to its cellular plasticity and increasing chemoresistance to current, clinically used anticancer drugs [3, 4]. The plethora of unwanted side effects of most chemotherapeutic drugs in use today and their declining effectiveness due to resistance mechanisms in cancer cells have made the discovery of more tolerable anticancer drugs, which are less susceptible to chemoresistance, incumbent and urgent. Natural products—in particular, plants—have been viable sources of chemical leads for anticancer drug development [5]. Random selection for screening, chemotaxonomic and pharmacophylogenetic relatedness to known sources of chemical leads, and ethnobotanical usage are some of the reasons why some plants are selected in screening campaigns for chemical leads identification in drug discovery programmes [6–10].

**Figure 1:**
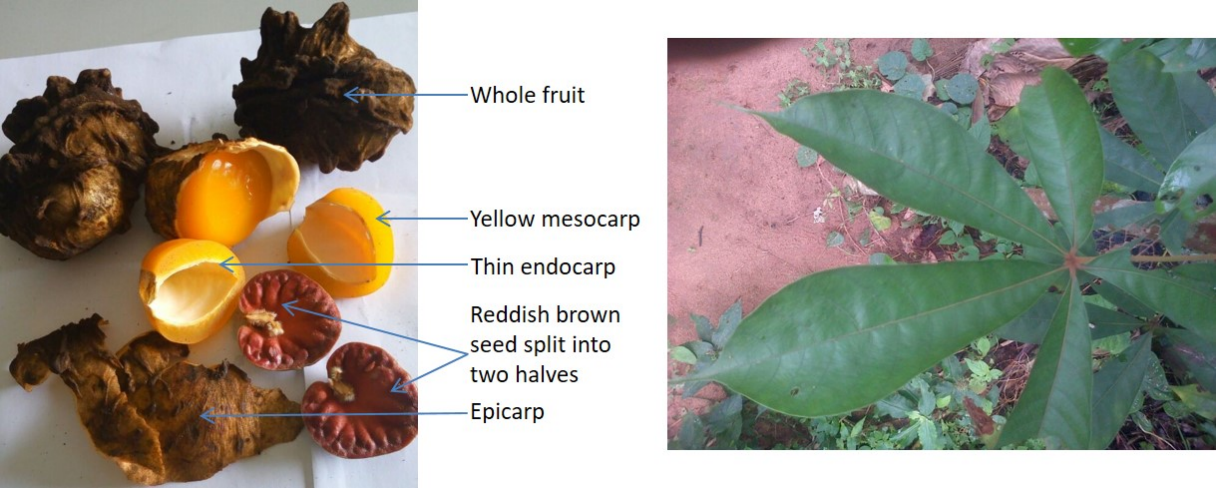
The fruits (left image) and leaves (right image) of *C. rostrata*

*Cola rostrata* K. Schum. is a tree crop of the Sterculiaceae family that is widely distributed in Gabon, southern Nigeria, and southwestern Cameroon [11–13]. *C. rostrata*, which has two varieties (yellow or white mesocarp) that grow mostly in the wild and sometimes in farms, produces fruits with crunchy, tasty, edible mesocarp [14–16]. The fruit of the yellow variety has a scaly, rough, yellowish, dark-brown epicarp covering usually one or two locules; the plant has 5–7 sub-sessile, broad, acuminate leaflets with a prominent midrib (Figure 1) [17].

Traditional use of its close varieties *C. lepidota* and *C. parchycarpa* for the treatment of cancer and pharmacological evidence of the cytotoxicity of their extracts against a panel of cancer cell lines have been reported [18, 19]. While there is limited information on the potential of *C. rostrata* as a source of anticancer compounds, its phylogenetic relatedness to *C. lepidota and C. parchycarpa* makes it a good candidate for investigation to ascertain its potential as a source of chemical leads for the development of anticancer drugs. Phytochemical analysis has revealed the presence alkaloids, saponins, terpenoids, flavonoids, phenols, steroids, tannins, cardiac glycosides and anthraquinones in parts of the plant [15, 20]. The fruit epicarp was reported to contain polyphenols, tannins and antimetabolites such as hydrocyanic acid, phytic acid and oxalic acids; and its extract inhibited the growth of *Escherichia coli*, *Staphylococcus aureus*, *β-haemolytic Streptococcus* and *Klebsiella pneumonia* [21]. A recent paper by the authors reported the presence of drug-like compounds in the fractions of ethanol extract of *C. rostrata* epicarp, which have protein targets that suggest the importance of the epicarp as a potential source of chemical agents for drug development for the treatment of diabetes, pain and inflammatory diseases, and cancer [22].

This study, therefore, examined the cytotoxic potential of ethanol extracts of the fruit epicarp and leaf of *C. rostrata*, using two human cell lines: a cervical cancer cell line, HeLa, and a transformed lung cell line, MRC5-SV2. The extracts were subjected to bioassay-guided fractionation, followed by elucidation of some of the mechanisms of cytotoxic action, and gas chromatography-mass spectrometry (GC-MS) analysis of one of the active fractions.

## Materials and methods

### Plant materials

Plant materials, the fruit and leaf samples, were obtained fresh from the trees at Utu Ikot-Essien, Ikot Ekpene Local Government Area of Akwa-Ibom State, southern Nigeria, between May and August 2018. The samples were authenticated by Professor Abiodun Ayodele of the Department of Botany, University of Ibadan, and the leaf sample specimen was deposited at the University of Ibadan Herbarium, with the voucher number UIH22970.

### Reagents and cell culture materials

Items and their sources included the following: Dulbecco’s Modified Eagle Medium (DMEM) (Thermo Fisher Scientific, UK); antibiotic-antimycotic (anti-anti) solution (Thermo Fisher Scientific, UK); foetal bovine serum (FBS; Sigma-Aldrich); L-glutamine (Thermo Fisher Scientific, UK); TrypLE (Thermo Fisher Scientific, UK); 3-(4,5-dimethylthiazol-2-yl)-2,5-diphenyltetrazolium bromide (MTT; Sigma-Aldrich); silica gel—pore size 60 Å 70–230 mesh 63–200 μm (Sigma-Aldrich); silica gel 60H (Merck, Germany); strata C18-E 55 μm, 70 Å 20 g/60 mL (Phenomenex); Dulbecco’s phosphate-buffered saline (D-PBS; Thermo Fisher Scientific, UK); 2ʼ,7ʼ-dichlorofluorescein diacetate (DCFDA); Reactive Oxygen Species (ROS) Assay Kit (Abcam); JC-1 solid (Sigma-Aldrich); black, microclear 96-well plates (Greiner Bio-One, UK).

### Preparation of extracts

*C. rostrata* leaves and fruit epicarp were air-dried separately under shade at ambient room temperature. The dried materials were cut into small pieces and pulverized with a blender. The leaf and epicarp materials were separately soaked in absolute ethanol for 6 days. The mixtures were vigorously shaken at regular intervals; the extracts were filtered and concentrated. The leaf extract was evaporated to dryness at 45℃, while the epicarp extract was air-dried in a regulated flow cupboard. The samples were labelled 2A and 1A, respectively.

### Cell culture

Immortalised human cervical adenocarcinoma (HeLa) and immortalised, SV40-transformed human lung fibroblast (MRC5-SV2) cells used in the study were originally obtained from the European Collection of Authenticated Cell Cultures (ECACC). The cells were grown as adherent monolayer cultures in T75 flasks or multi-well plates using, as growth medium, DMEM (with phenol red) containing 4.5 g/L glucose, supplemented with 10% FBS, 2 mmol/L L-glutamine, and 1% antibiotic-antimycotic solution was used. The cells were cultured in T75 flasks and incubated in a humidified atmosphere of 37℃ and 5% CO_2_; they were prepared and used as described by Omondi et al. [23].

### Treatment of cells with extracts

HeLa and MRC5-SV2 cells were separately prepared at densities 1 × 10^5^ cells/mL, 7.5 × 10^4^ cells/mL and 5 × 10^4^ cells/mL and seeded into 96-well plates (100 μL per well) for 24 h, 48 h and 72 h treatments, respectively. Stock solution (50 mg/mL) of each of the two crude extracts was prepared in dimethyl sulfoxide (DMSO). The seeded cells were allowed to attach for 24 h and then treated with the extracts, up to 72 h, at final concentrations of 10, 50, 100 and 200 μg/mL, each in triplicate. The final percentage of DMSO (v/v) in the growth medium was less than 0.4%, which was not toxic to the cells. Each treatment was run at least three independent times. Cell morphology changes induced by the treatments were viewed with an Olympus CKX41 microscope fitted with an Olympus DP71 U-TVIX-2 camera and the images were captured with the Olympus cellSens entry 1.16 imaging software.

### Cell viability assay

Cell viability was assessed using the MTT assay as previously reported [23]. To each treatment well in the 96-well plate (clear, flat bottom), 10 μL of a 5 mg/mL MTT solution prepared in phosphate-buffered saline (PBS; pre-warmed to 37℃) was added. The cells were then incubated for 2 h to allow them to convert the MTT into purple-coloured formazan. Afterwards, the liquid content of each well was carefully aspirated to leave only the formazan crystals in the wells. DMSO (100 μL) was added to each of the wells to dissolve the crystals. The plates were shaken on a rotary shaker for approximately 5 min to ensure complete dissolution of the crystals and to achieve a homogeneous solution. Absorbance was then read at 570 nm using the CLARIOstar microplate reader (BMG LABTECH, UK).

### Fractionation of extracts

Extracts 1A and 2A were subjected to solvent partitioning using a separatory funnel. 1A (0.86 g) was partitioned in 60 mL 70% methanol and 40 mL *n*-hexane; the sub-fractions were concentrated under nitrogen using a techne sample concentrator which was regulated to 45℃ and air-dried. The polar sub-fraction was labelled 1AM, while the non-polar sub-fraction was labelled 1AFK. 2A (8.74 g) was partitioned in two batches between 70% methanol and *n*-hexane; each batch had a total volume of 300 mL in a ratio 2:1 (70% methanol:*n*-hexane). The polar sub-fraction was concentrated under nitrogen, air-dried and labelled 2AFM. The non-polar sub-fraction had a component which partitioned with it but remained undissolved; the sub-fraction was filtered and the filtrate was concentrated, air-dried and labelled 2AFH. The undissolved fraction was found to be soluble in ethyl acetate, and the sub-fraction obtained therefrom was filtered, air-dried and labelled 2AFU.

**Figure 2:**
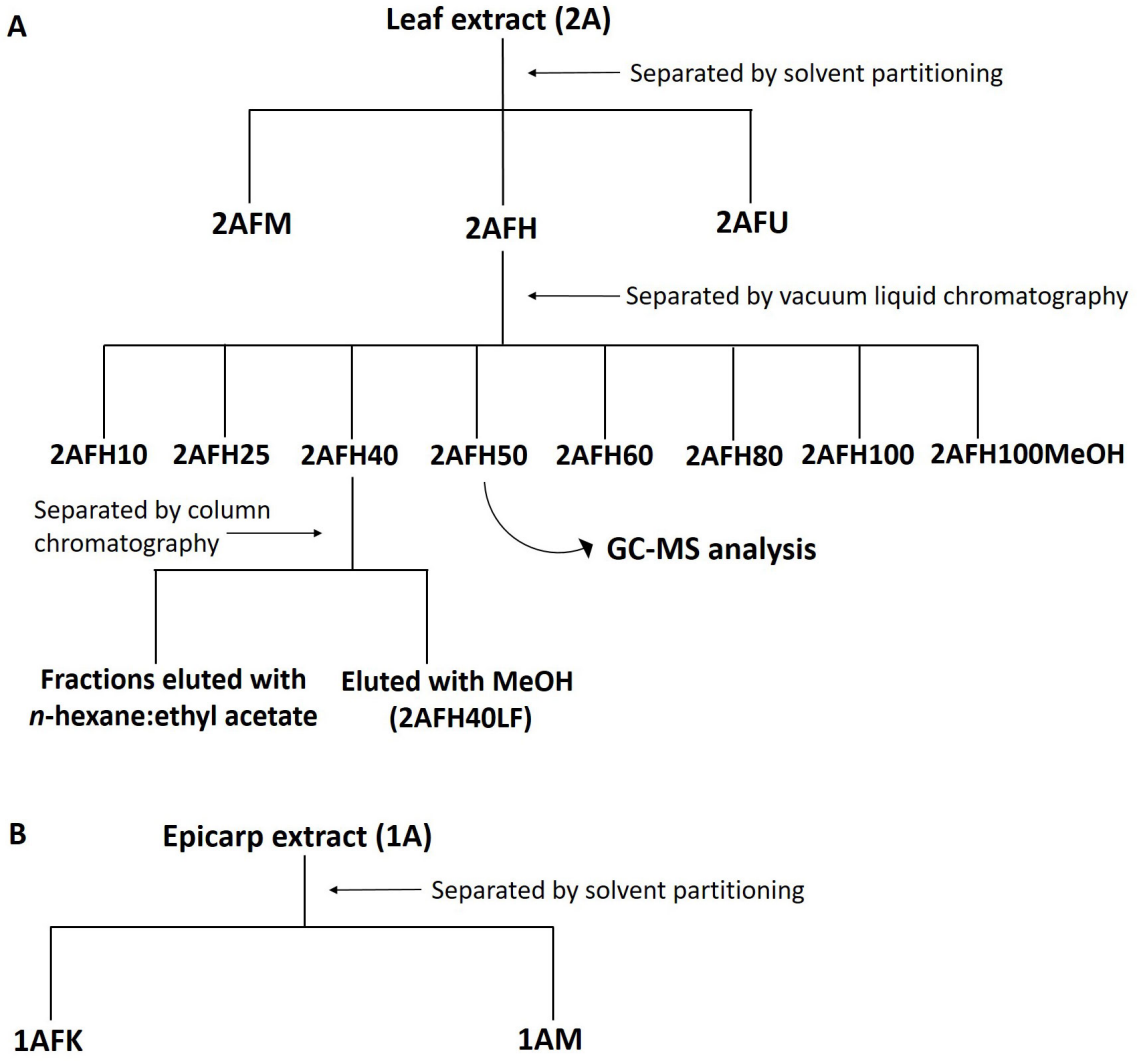
Fractionation scheme for ethanol extracts of *C. rostrata* leaf and fruit epicarp. (A) The leaf extract, 2A, had fractions separated by solvent partitioning and chromatographic methods; (B) the epicarp extract, 1A, was separated by solvent partitioning into polar and non-polar fractions

Based on the cytotoxicity assays, 2AFH (1.5 g) was separated using vacuum liquid chromatography (VLC) and eluted with a mobile phase composed of varying proportions of *n*-hexane and ethyl acetate: 100% *n*-hexane, 95:5, 90:10, 75:25, 60:40, 50:50, 40:60, 20:80 and 100% ethyl acetate; the fraction left in the column was eluted with 100% methanol. The 100% *n*-hexane and 5% ethyl acetate in *n*-hexane did not elute any significant amount of the extract, and the remaining sub-fractions were labelled 2AFH10, 2AFH25, 2AFH40, 2AFH50, 2AFH60, 2AFH80, 2AFH100 and 2AFH100MeOH, respectively. 2AFH40 was further separated using column chromatography and eluted with a mobile phase comprising various ratios of *n*-hexane and ethyl acetate from 100% *n*-hexane to 100% ethyl acetate. The fractionation schemes for crude extracts 1A and 2A are shown in Figure 2.

### DCFDA cellular ROS assay

To determine aspects of the mechanisms of cytotoxicity of the extract fractions, potential generation of intracellular ROS was assessed using the DCFDA Cellular ROS Detection Assay Kit (Abcam, catalogue number ab113851) according to the manufacturer’s protocol and as previously described [23]. Each extract fraction was dissolved in DMSO and tested on HeLa cells cultured 24 h previously at a density of 2.5 × 10^5^ cells/mL (100 μL/well in a black, microclear 96-well plate). The final concentrations of each extract fraction tested were 10, 50, 100 and 200 μg/mL (prepared in growth medium without phenol red). The concentrations of the positive control [tert-butyl hydroperoxide (TBHP)] were 100 μmol/L and 200 μmol/L. Fluorescence at 483-14 nm/530-30 nm was recorded from 3 h up to 24 h post-treatment, using the CLARIOstar microplate reader (BMG LABTECH, UK). Background fluorescence was subtracted from each value before fold increase in fluorescence intensity relative to the negative control was determined. The assay was conducted three independent times.

### Mitochondrial membrane depolarization assay (JC-1 staining)

MRC5-SV2 cells were seeded into a 24-well Falcon plate at a density of 1 × 10^5^ cells/mL and a volume of 300 μL/well. The cells were allowed to attach for 24 h and then examined under the microscope to confirm their attachment and healthy, uncontaminated growth. 2-fold (2×) solutions of 10, 50, 100 and 200 μg/mL of 1AFK, 1AM, 2AFH25 and 2AFH50 were prepared from their stock solutions (50 mg/mL in DMSO) using pre-warmed growth medium. From each well, 150 μL of growth medium was removed and 150 μL of each 2× concentration of each extract fraction was added. Fresh growth medium (150 μL) was added to two wells which served as the growth medium control wells, and 150 μL of growth medium containing DMSO in the same percentage of DMSO (v/v) as it was in the highest test concentration (200 μg/mL) was added to two wells which served as the DMSO control wells. The cells were incubated at 37℃ for 3 h.

A 1:200 dilution of a 4 mmol/L stock 5,5ʼ,6,6ʼ-tetrachloro-1,1ʼ,3,3ʼ-tetraethyl-imidacarbocyanine iodide (JC-1) solution in DMSO was prepared in pre-warmed growth medium. Working JC-1 solution (30 μL) was added to each well (final concentration 2 μmol/L) and the cells were incubated for 1 h at 37℃. The medium in each well (corresponding to a concentration of each test fraction) was aspirated into a labelled Eppendorf tube to collect all suspended cells. PBS (200 μL) was used to rinse each well to remove residual growth medium. The PBS was then carefully aspirated, 100 μL of TrypLE was added to detach the adherent cells, and the cells were incubated at 37℃ for 5 min for complete detachment. Fresh growth medium (100 μL) was added to each well to stop the trypsin action; the content of each well was collected into the corresponding Eppendorf tube already containing the suspended cells, and the content was mixed. Fluorescence was determined using a BD Accuri C6 flow cytometer. Morphologically viable and dying cells were gated based on forward and side scatter parameters and to the exclusion of debris. Gated cells were then analysed for monomeric JC-1 green fluorescence (FL1: λ_ex_ 488 nm; λ_em_ 533/30 nm) and polymeric yellow/orange fluorescence (FL2: λ_ex_ 488 nm; λ_em_ 585/25 nm). Compensation for FL1 spillover into FL2 was performed and set at 10.54% and a total of 5,000 cell events were collected for each sample. Data was analysed using the instrument’s C6 Analysis software.

### GC-MS analysis of active fraction

Fraction 2AFH50 was analyzed on Gas Chromatography (Agilent Technologies, 7890, USA) apparatus coupled with a Mass Spectrometer (Agilent Technologies, 5975, USA). The column (Agilent Technologies, HP5MS) had a length of 30 cm, internal diameter of 0.320 mm and thickness of 0.25 μm. The initial oven temperature was set to 80℃ for 2 min, increased at a rate of 12℃ per min to 240℃ and then held for 6 min. The volume injected was 1 μL and the mode of analysis was splitless. The interface temperature between the Gas Chromatography apparatus and the mass spectrometer was 250℃. The total run time was 22 min and scans ranged from 50 m/z to 500 m/z, and a search of the National Institute of Standards and Technology (NIST) 14 library was used to identify the compounds represented by the peaks.

### Data presentation and statistical analysis

Experiments were conducted at least three independent times, with each treatment for each run assessed at least in duplicate. Except otherwise stated, each experimental value is presented as mean ± standard error of the mean (SEM). One-way analysis of variance (ANOVA) followed by post-hoc test (Tukey test) for multiple comparisons was used to determine statistical significance of differences between means, using the GraphPad Prism software (Version 8.0.1; GraphPad Software Incorporated, CA, USA). A *P*-value < 0.05 was considered statistically significant. IC_50_ value was determined using GraphPad Prism by fitting the data to the non-linear regression “log [inhibitor] *versus* normalized response”.

## Results

### Cytotoxicity of crude extracts

**Figure 3:**
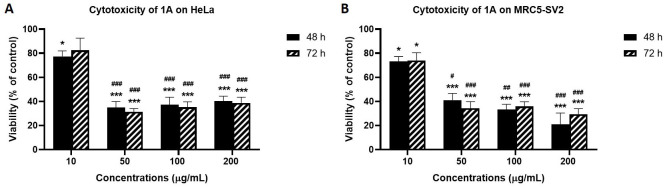
Effects of 48 h and 72 h treatments with extract 1A on the viability of (A) HeLa cells, and (B) MRC5-SV2 cells. ^*^ *P* < 0.05 and ^***^ *P* < 0.001 represent significant differences in cell viability between 1A treatment and negative control, while ^#^ *P* < 0.05, ^##^ *P* < 0.01 and ^###^ *P* < 0.001 represent significant differences in cytotoxicity between the indicated concentration and 10 μg/mL treatment. The experiment was run three independent times (*n* = 3)

**Figure 4:**
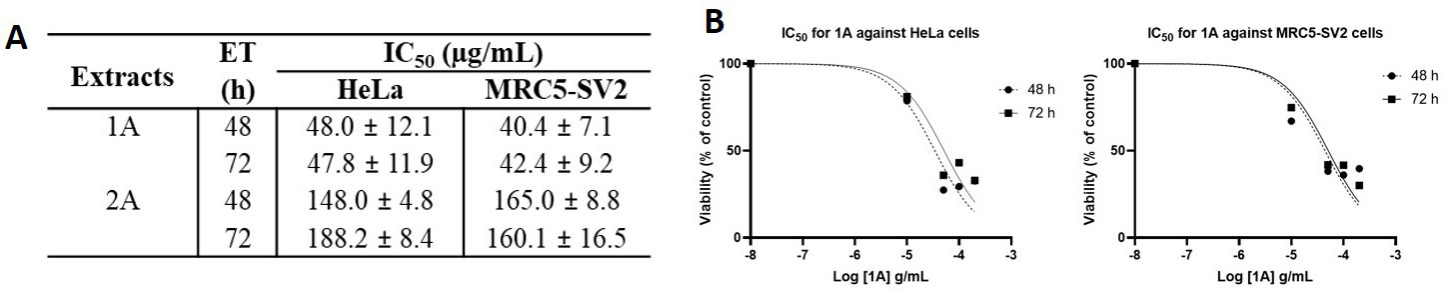
Cytotoxic effects of 1A and 2A on HeLa and MRC5-SV2 cells. (A) IC_50_ values for the cytotoxicity of 1A and 2A against HeLa and MRC5-SV2 cells; (B) representative concentration-response curves for the cytotoxicity of 1A against HeLa and MRC5-SV2 cells at 48 h and 72 h of treatment. ET: exposure time in hours

As shown in Figure 3 that depicts the effects of the epicarp extract 1A and Figure 4 that lists the IC_50_ values of 1A and the leaf extract 2A, both 1A and 2A were cytotoxic against HeLa and MRC5-SV2 cells when tested within the concentration range 10–200 μg/mL for up to 72 h. However, the effects were not exposure time-dependent, as most values at 72 h were not significantly different to values at 48 h (Figures 3 and 4). The cytotoxic effect of 1A against both cell lines was significant from 10 μg/mL and concentration-dependent across 10 μg/mL and 50 μg/mL but then peaked at 50 μg/mL for the HeLa cells (Figure 3A), while generally further decreases in viability beyond that elicited by 50 μg/mL were modest (48 h) or insignificant (72 h) in the MRC5-SV2 cells (Figure 3B). Over both time points (48 h, 72 h) and against the two cell lines, 1A was approximately three to four times more potently cytotoxic than 2A (Figure 4). There was evidence that morphological damage accompanied the cytotoxicity induced by the extracts, as their higher concentrations caused loss of confluency and shrinkage of most of the remaining cells.

### Cytotoxicity of fractions

Solvent partitioning of 2A yielded three fractions: 2AFH, 2AFM and 2AFU; 2AFH and 2AFU each showed significant activity at most of the four concentrations tested, while 2AFM only showed significant cytotoxic (*P* < 0.001) activity at 200 μg/mL against HeLa and MRC5-SV2 cells at 48 h and 72 h time points (Table 1). 2AFH and 2AFU showed concentration-dependent cytotoxicity against both cell lines in all the concentrations tested at 48 h, with significant difference (*P* < 0.001) between the viabilities of the treated wells and those of the negative control wells (Table 1). At the lowest concentration tested (10 μg/mL), surprisingly the 72 h treatment of both cell lines with 2AFH and 2AFU, as opposed to the 48 h treatment, did not reduce the viability of the cells in most cases. The IC_50_ values for the cytotoxicities of the three fractions are also shown in Table 1. The effects of 2AFH and 2AFU were generally not time-dependent against both cell lines. In contrast, 2AFM was more cytotoxic against the HeLa cell line in the longer (72 h) time point.

**Table 1:** Cytotoxicity of 2A fractions: 2AFH, 2AFM and 2AFU against HeLa and MRC5-SV2 cells

**2A Fractions**	**Cell line**	**ET (h)**	**Cell viability (negative control normalised to 100%)**				**IC_50_ (μg/mL)**
			**10 μg/mL**	**50 μg/mL**	**100 μg/mL**	**200 μg/mL**	
2AFH	HeLa	48	72.3 ± 2.5^***^	48.7 ± 3.0^***^	39.0 ± 4.9^***^	32.7 ± 4.7^***^	59.4 ± 11.1
		72	104.6 ± 3.3	59.6 ± 2.8^***^	40.3 ± 3.6^***^	31.2 ± 1.2^***^	84.0 ± 6.1
	MRC5-SV2	48	80.4 ± 5.1^*^	55.3 ± 4.3^***^	42.1 ± 3.6^***^	33.3 ± 4.0^***^	63.2 ± 12.4
		72	104.3 ± 3.9	49.4 ± 6.6^***^	30.3 ± 5.3^***^	21.5 ± 3.5^***^	60.4 ± 11.8
2AFM	HeLa	48	120.6 ± 4.3	120.8 ± 6.8	109.0 ± 2.4	48.2 ± 7.5^***^	> 700
		72	106.7 ± 5.8	111.4 ± 2.0	78.9 ± 6.5	27.5 ± 3.0^***^	235.0 ± 17.8
	MRC5-SV2	48	105.0 ± 3.5	115.4 ± 3.7	98.9 ± 5.4	46.6 ± 11.8^***^	> 500
		72	136.3 ± 15.2	112.8 ± 1.1	100.5 ± 4.2	63.0 ± 3.1^***^	> 600
2AFU	HeLa	48	74.8 ± 2.0^***^	60.9 ± 2.7^***^	50.1 ± 4.1^***^	41.3 ± 4.3^***^	99.0 ± 14.2
		72	79.6 ± 4.6^**^	63.0 ± 3.4^***^	49.5 ± 3.3^***^	30.2 ± 3.0^***^	87.0 ± 10.7
	MRC5-SV2	48	74.3 ± 3.1^***^	62.1 ± 2.7^***^	54.5 ± 1.9^***^	43.8 ± 0.9^***^	109.0 ± 12.2
		72	106.9 ± 4.4	76.7 ± 1.7^*^	56.5 ± 4.1^***^	38.5 ± 4.6^***^	146.0 ± 17.5

^*^ *P* < 0.05, ^**^ *P* < 0.01, and ^***^ *P* < 0.001 indicate a significant difference between the cell viability of treated cells and the cell viability of untreated cells of the negative control wells. ET: exposure time in hours

**Figure 5:**
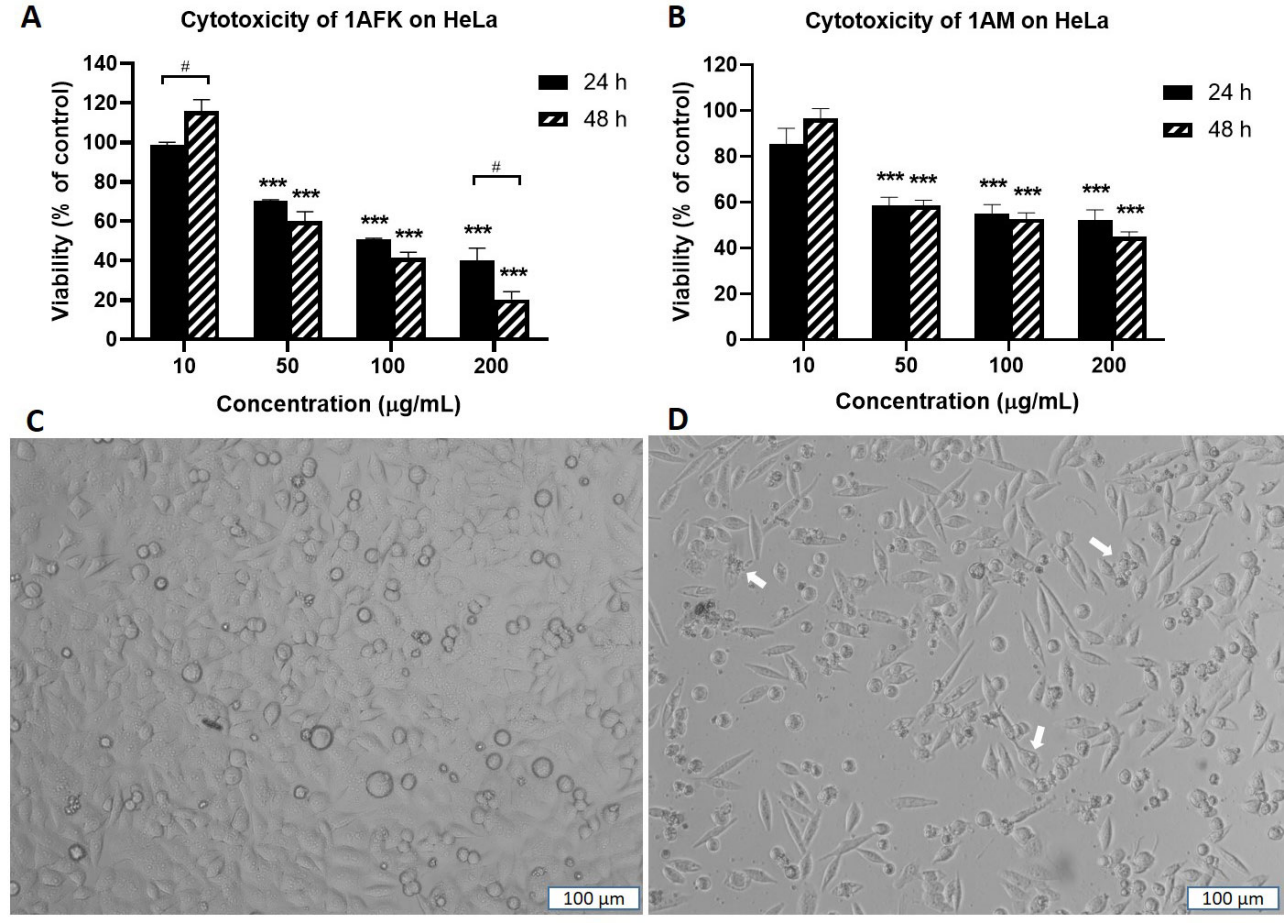
Cytotoxicity of fractions 1AFK and 1AM against HeLa cells after 24 h and 48 h treatments. Viability of cells in untreated wells (negative control) was taken as 100%. (A) HeLa cells treated with 1AFK; (B) HeLa cells treated with 1AM; (C) photomicrograph of negative control HeLa cells after 24 h treatment; (D) photomicrograph of HeLa cells treated with 200 μg/mL of 1AFK for 24 h, showing reduced cell number. White arrows point to dying cells with morphological features consistent with apoptosis. ^***^ *P* < 0.001 represents a significant difference in cell viability between negative control cells and cells treated with fraction; ^#^ *P* < 0.05 represents a significant difference between 24 h cytotoxicity and 48 h cytotoxicity of the same concentration. Magnification: ×100, scale bar: 100 μm

Solvent partitioning of 1A gave two fractions: 1AFK (non-polar) and 1AM (polar). 1AFK showed greater cytotoxicity than 1AM on both cell lines, especially at 48 h (Figure 5A and B for HeLa). The photomicrographs (Figure 5C and D) show a reduction in HeLa cell number when the negative control cells (Figure 5C) are compared with the 1AFK (200 μg/mL)-treated cells (Figure 5D), as well as morphological changes to the cells following 24 h treatment with 1AFK. At higher concentrations of 1AFK, dying cells had morphological features consistent with apoptosis, including shrinkage (Figure 5D, see white arrows). 1AFK elicited concentration-dependent and time-dependent cytotoxicity against both cell lines. 1AM showed significant cytotoxicity (*P* < 0.001) against HeLa cells from the 50 μg/mL treatment up to the 200 μg/mL treatment for both 24 h and 48 h. The cytotoxicity of 1AM was, however, generally not significantly concentration-dependent or time-dependent against HeLa cells (Figure 5B); and it was not concentration-dependent against MRC5-SV2 cells after 48 h (figures not shown). The IC_50_ values for the cytotoxicity of 1AFK against HeLa and MRC5-SV2 cells are 122.0 μg/mL ± 12.0 μg/mL and 132.0 μg/mL ± 24.4 μg/mL, respectively, for the 24 h treatments; and 81.0 μg/mL ± 12.7 μg/mL and 52.2 μg/mL ± 3.5 μg/mL, respectively, for the 48 h treatments. For 1AM, the IC_50_ values for its cytotoxicity are 134.0 μg/mL ± 26.4 μg/mL and 114.0 μg/mL ± 12.7 μg/mL for 24 h and 48 h treatments of HeLa cells, respectively; and 80.1 μg/mL ± 4.3 μg/mL for 48 h treatment of MRC5-SV2 cells.

### Cytotoxicity of 2AFH fractions

**Figure 6:**
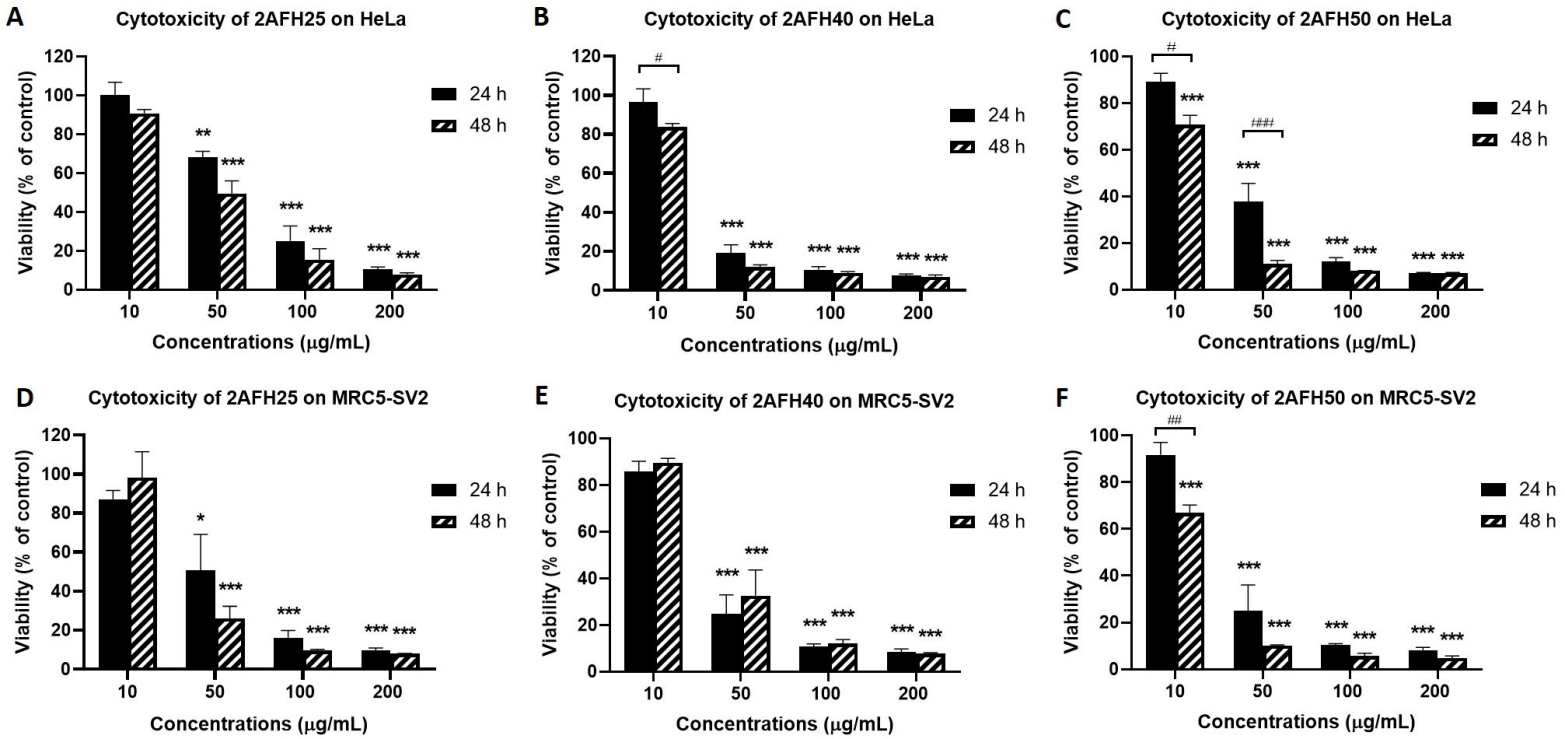
Cytotoxicity of some 2AFH fractions against HeLa and MRC5-SV2 cells after 24 h and 48 h treatments. (A, D) Cytotoxicity of 2AFH25 against HeLa and MRC5-SV2 cells; (B, E) cytotoxicity of 2AFH40 against HeLa and MRC5-SV2 cells; (C, F) cytotoxicity of 2AFH50 against HeLa and MRC5-SV2 cells. ^*^ *P* < 0.05, ^**^ *P* < 0.01 and ^***^ *P* < 0.001 represent a significant difference in cell viability between treatment with fraction and treatment with negative control; ^#^ *P* < 0.05, ^##^ *P* < 0.01 and ^###^ *P* < 0.001 represent a significant difference between 24 h cytotoxicity and 48 h cytotoxicity of the same concentration of the fraction

VLC fractionation of 2AFH yielded eight fractions (see Figure 2) with varying levels of cytotoxicity. The yellowish fraction, 2AFH10, was not tested because it was only sparingly soluble in DMSO. 2AFH25 and 2AFH40 were moderately soluble in DMSO when prepared as 50 mg/mL stock solutions, while the remaining fractions were fully soluble. The concentration-dependent (and, in some cases, time-dependent) cytotoxic effects of 2AFH25, 2AFH40 and 2AFH50 are shown in Figure 6 and the IC_50_ values for the seven fractions (apart from the insoluble 2AFH10) are shown in Table 2. 2AFH60 was nearly equipotent against both cell lines, with 24 h cytotoxicity IC_50_ values of 24.6 μg/mL ± 2.1 μg/mL and 26.4 μg/mL ± 4.7 μg/mL, for HeLa and MRC5-SV2 cells, respectively. 2AFH80 and 2AFH100 significantly reduced the viability of MRC5-SV2 cells, with IC_50_ values of 16.4 μg/mL ± 0.6 μg/mL and 30.9 μg/mL ± 3.6 μg/mL, respectively, after 48 h treatment. 2AFH-derived fractions were generally more toxic than the parent extract. Among the fractions, there was a gradual reduction in cytotoxicity as polarity increased (Table 2). It should be noted that the mass of 2AFH50 obtained was relatively small compared to that of 2AFH40, and 2AFH50 was, therefore, not considered suitable for further fractionation.

**Table 2:** IC_50_ values for the cytotoxic effects of 2AFH fractions on HeLa and MRC5-SV2 cells following 24 h and 48 h treatments (*n* = 3)

**2AFH fractions**	**HeLa (IC_50_, μg/mL)**		**MRC5-SV2 (IC_50_, μg/mL)**	
	**24 h**	**48 h**	**24 h**	**48 h**
2AFH25	60.9 ± 7.7	39.6 ± 7.2	41.8 ± 12.0	27.9 ± 4.3
2AFH40	25.5 ± 3.4	18.9 ± 0.4	24.6 ± 2.8	26.7 ± 4.7
2AFH50	31.3 ± 4.4	14.9 ± 0.8	26.4 ± 4.5	12.8 ± 1.0
2AFH60	24.6 ± 2.1	18.5^a^	26.4 ± 4.7	15.9 ± 0.9
2AFH80	24.3^a^	27.0 ± 6.9^b^	22.6 ± 3.1^b^	16.4 ± 0.6
2AFH100	83.2 ± 3.6	35.4 ± 1.2	47.5 ± 1.5	30.9 ± 3.6
2AFH100MeOH	ND	104.0 ± 15.2	ND	80.4 ± 11.2

ND: not determined. ^a^ value is for *n* = 1, and ^b^ value is for *n* = 2

### Effects on intracellular levels of ROS

**Figure 7:**
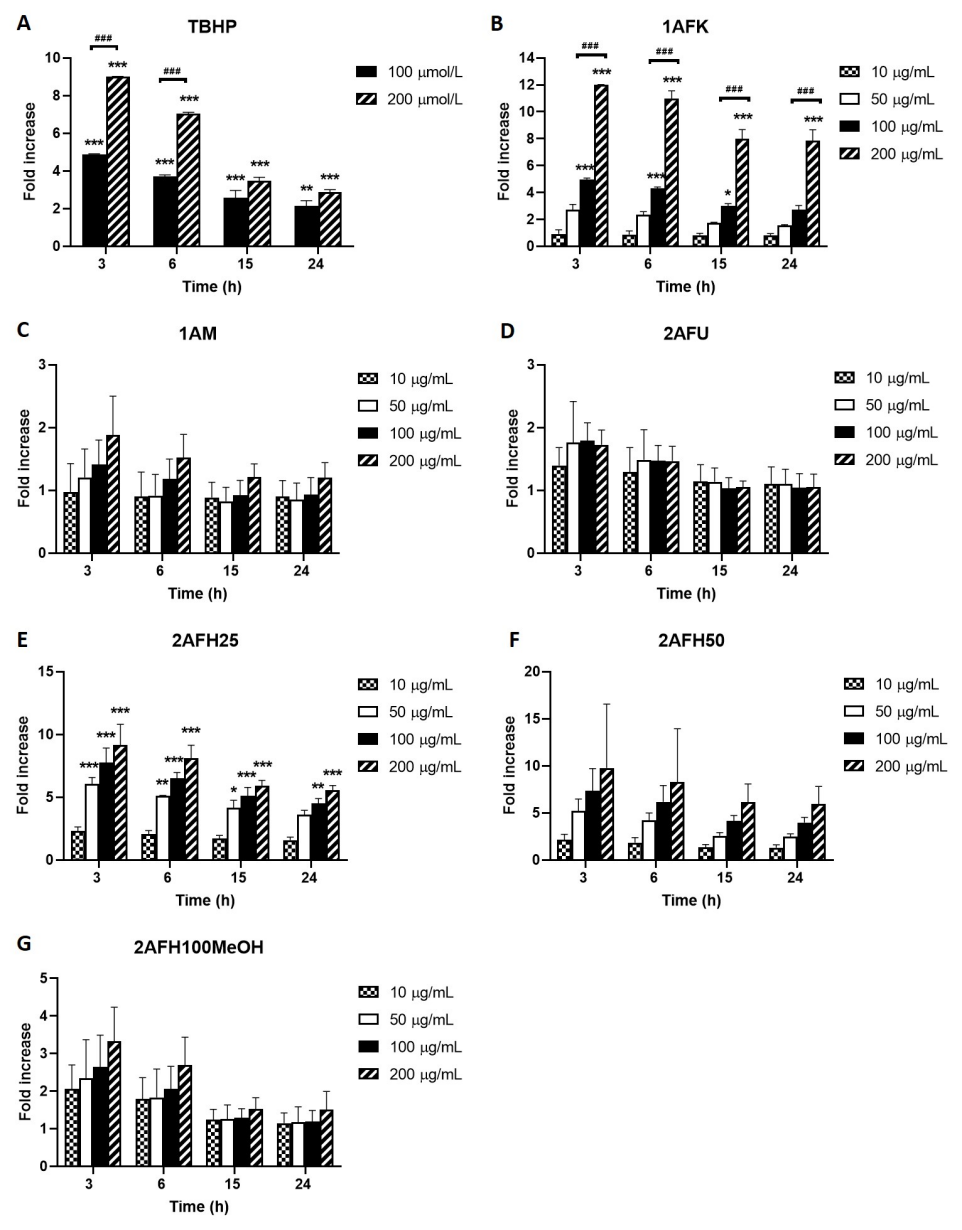
Intracellular ROS generation by extract fractions in HeLa cells over a 24 h period (expressed as fold increases compared to the negative control). (A) TBHP (positive control); (B) 1AFK; (C) 1AM; (D) 2AFU; (E) 2AFH25; (F) 2AFH50; (G) 2AFH100MeOH. ^*^ *P* < 0.05, ^**^ *P* < 0.01 and ^***^ *P* < 0.001 represent a significant difference in intracellular ROS generation between treatment and negative control; ^###^ *P* < 0.001 represents a significant difference in ROS generation between indicated concentrations of the same extract fraction at the same time point. Each experiment was run three independent times (*n* = 3)

ROS generation in HeLa cells due to exposure to different concentrations of 1AFK, 1AM, 2AFU, 2AFH25, 2AFH50 and 2AFH100MeOH was determined in a bid to identify potential mechanisms of action of the secondary metabolites in the extract fractions that might underlie their cytotoxicity and induction of cell death. TBHP, as positive control, induced concentration-dependent increases in ROS levels at 3, 6, 15 and 24 h exposure time points, peaking at 3 h time point, where at 100 μmol/L and 200 μmol/L it induced 5-fold and 9-fold increases, respectively (Figure 7A). 1AFK induced ROS in a concentration-dependent manner at each of the four time points examined, with 200 μg/mL at 3 h producing nearly a 12-fold increase (Figure 7B).

1AM induced apparently concentration-dependent but largely insignificant ROS production, which at the highest concentration (200 μg/mL) was approximately 2-fold increase for the 3 h time point, an effect that progressively dissipated over the 24 h time period (Figure 7C). The intracellular ROS-generating activity of 2AFU, as depicted in Figure 7D, was not concentration-dependent; at its peak ROS production time point (3 h), the 50 μg/mL, 100 μg/mL and 200 μg/mL treatments each produced an average of about 1.7-fold increase in ROS levels, which progressively reduced to near negative control levels within 15 h following treatment.

2AFH25 and 2AFH50 each induced a concentration-dependent generation of cellular ROS that peaked within 3 h of exposure to them (Figure 7E and F). At the lowest concentration, 10 μg/mL, 2AFH25 and 2AFH50 each produced about a 2-fold increase in ROS levels within 3 h, and at the highest concentration, 200 μg/mL, 2AFH25 produced a 9-fold increase, while 2AFH50 produced a 9.7-fold increase (there was wide variability for 2AFH50 effect). At 24 h, the ROS levels maintained by 2AFH25 and 2AFH50 were about 5.5-folds and 6.4-folds, respectively. 2AFH100MeOH induced concentration-dependent increases in cellular ROS at the 3 h and 6 h time points, beyond which the levels were not different to those of negative control cells (Figure 7G).

### Effects on mitochondrial membrane integrity

HeLa cells were treated with five extract fractions: 1AFK, 1AM, 2AFH25, 2AFH50 and 2AFH40LF at final concentrations of 10, 50, 100 and 200 μg/mL over a 4 h period. 2AFH40LF, the major fraction derived from 2AFH40, was introduced here and in subsequent mechanistic assays in order to compare or contrast the effect of such a downstream fraction with those of the first- and second-level fractions (the IC_50_ for the cytotoxicity of 2AFH40LF against HeLa cells after 24 h was 55.1 μg/mL ± 2.7 μg/mL). The fluorescence of JC-1, a cationic dye which diffuses easily across mitochondrial membrane and accumulates in the negatively charged mitochondrial matrix, was used as a readout for the mitochondrial membrane potential (∆Ψ_M_), which is an index of mitochondrial membrane integrity (mitochondrial health). JC-1 molecules reversibly form aggregates (polymers) in the mitochondria of healthy cells and predominance of the monomers in the mitochondria is indicative of impaired mitochondrial function and initiation of the apoptotic cell death process. The ratio of the count of JC-1 polymers (aggregates) that give high yellow/orange fluorescence and detect healthy cells, to the count of JC-1 monomers that give higher green fluorescence and detect apoptotic cells, was evaluated as a surrogate for ∆Ψ_M_.

**Figure 8:**
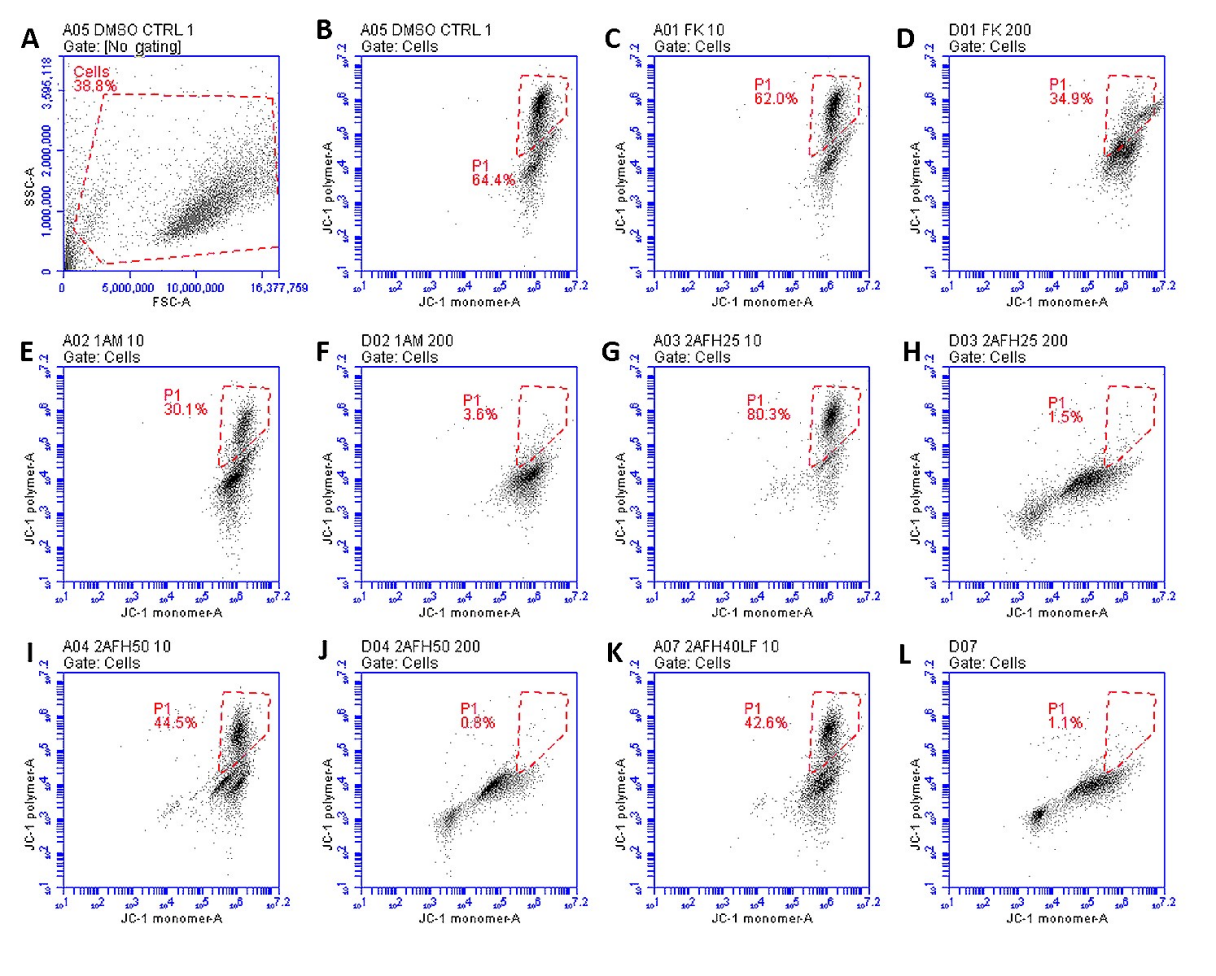
Representative density plots of JC-1 staining of HeLa cells following treatment with extract fractions 1AFK (indicated as “FK”), 1AM, 2AFH25, 2AFH40LF and 2AFH50 at 10 μg/mL and 200 μg/mL (numerals after each extract designation indicate concentrations in μg/mL). P1 represents cells containing polarized mitochondria. (A, B) Plots for the negative control; (C-F) plots of 1A fractions showing no pattern of early and late apoptotic cell populations; (G-L) plots of 2AFH fractions showing a pattern of early and late apoptotic cell populations

**Figure 9:**
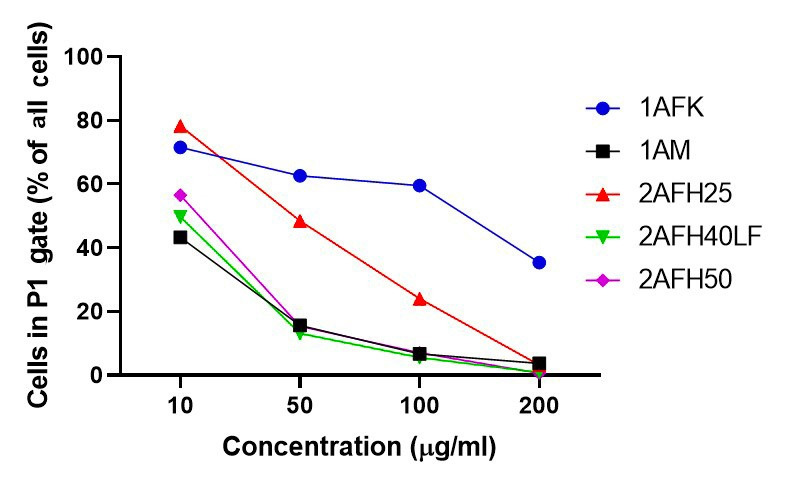
Average proportions of HeLa cells in P1 gate after 4 h treatment with extract fractions 1AFK, 1AM, 2AFH25, 2AFH40LF and 2AFH50 and staining with JC-1

The distribution and count of cells stained with JC-1 which appeared as monomers and polymers for each treatment are displayed in Figure 8. Firstly, Figure 8A shows all sample events in a negative control and the gating, based on forward and side scatter, to eliminate debris and select morphologically viable and apoptotic “cells” for assessment of JC-1 fluorescence. P1, as shown in Figure 8B, represents JC-1-labelled viable cells which have both high green and yellow/orange fluorescence. The population of cells outside of P1 represents cells with depolarized mitochondria and, thus, little polymeric JC-1 (yellow/orange fluorescence). The density plots for 10 μg/mL and 200 μg/mL concentrations of the five extract fractions are shown in Figure 8C–L, with 2AFH25, 2AFH40LF and 2AFH50 showing similar patterns of concentration-dependent change, though with different proportions of cells gated in P1. Several plots also depict two populations of depolarized cells with differing levels of green fluorescence, indicative of disrupted JC-1 dye loading into cells and mitochondria. The proportion of cells in P1 area for each of the extract fractions shows a concentration-dependent reduction in population (Figure 9). Based on the proportion of HeLa cells in P1, extract fractions 1AM, 2AFH40LF and 2AFH50 appear to be more efficient at disrupting mitochondrial function even at a concentration as low as 10 μg/mL within 4 h of treatment.

**Figure 10:**
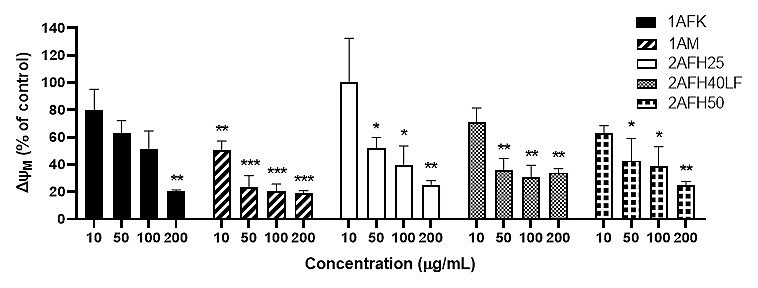
Effects of extract fractions 1AFK, 1AM, 2AFH25, 2AFH40LF and 2AFH50 on the ∆Ψ_M_ in HeLa cells. Each value is expressed as a percentage of the ∆Ψ_M_ for the negative control. ^*^ *P* < 0.05, ^**^ *P* < 0.01 and ^***^ *P* < 0.001 compared to the negative control

The plot of ∆Ψ_M_ (as a percentage of the ∆Ψ_M_ for the negative control) *versus* concentration, following treatment with each of the five extract fractions, is shown in Figure 10. All extract fractions reduced Ψ_M_ in a concentration-dependent manner. With respect to 1A fractions, 1AFK reduced Ψ_M_ significantly at 200 μg/mL (*P* < 0.01), while 1AM caused significant reductions (*P* < 0.01 or *P* < 0.001) at all of its concentrations. With regard to the 2AFH fractions 2AFH25, 2AFH40LF and 2AFH50, each showed a significant reduction (*P* < 0.05 or *P* < 0.01) in Ψ_M_ at each of its concentrations tested, except 10 μg/mL.

### Components of 2AFH50 identified by GC-MS

GC-MS identified 16 compounds in 2AFH50, with the most abundant components being fatty acids with retention times ranging between 9.930 min and 21.462 min. Based on proportion in the fraction, the top five components are *n*-hexadecanoic acid (52.29%), decanoic acid 10-(2-hexylcyclopropyl) (10.78%), octadecanoic acid (8.48%), 24S-Ethyl-5.alpha.-cholesta-2, 22E-dien-6-one (5.26%), and stigmasta-3,5-dien-7-one (3.24% + 2.17%). The components of 2AFH50 identified by GC-MS are shown in Table 3.

**Table 3:** Compounds identified in fraction 2AFH50 via GC-MS

**Components of 2AFH50**	**RT (min)**	**Qual %**	**Area %**	**MM (g/moL)**
2,4-Di-*tert*-butylphenol	9.930	94	1.05	206.32
Tetradecanoic acid	12.808	97	1.59	228.37
2-Pentadecanone, 6,10,14-trimethyl-	13.585	80	1.29	268.50
*n*-Hexadecanoic acid	14.996	99	52.29	256.40
Heptadecanoic acid	15.763	91	1.42	270.45
13-Tetradece-11-yn-1-ol	16.107	59	1.37	208.34
1,6-Dimethyl-9-(1-methylethylidene)-5,12-dioxatricyclo[9.1.0.0(4,6)]dodecan-8-one	16.420	49^a^	1.63	250.33
Octadecanoic acid	16.696	99	8.48	284.48
2,2,4a,6a,8a,9,12b,14a-Octamethyl-1,2,3,4,4a,5,6,6a,6b,7,8,8a,9,12,12a,12b,13,14,14a,14b-eicosahydropicene	17.218	46^a^	1.58	410.70
Decanoic acid, 10-(2-hexylcyclopropyl)	17.429	99	10.78	296.49
1,4-Dioxaspiro[4.5]decane-6-carboxylic acid, dimethylamide	18.218	89	2.10	213.27
Cholesta-8,24-dien-3-ol, 4-methyl-, (3.beta.,4.alpha.)-	19.174	90	1.20	398.70
A-Norcholestan-3-one, 5-ethenyl-, (5.beta.)-	19.251	41^a^	2.47	398.50
24S-Ethyl-5.alpha.-cholesta-2,22E-dien-6-one	20.784	64	5.26	410.70
Stigmasta-3,5-dien-7-one	21.273	70	5.41	410.68
2H-3,9a-methano-1-benzoxepin, octahydro-2,2,5a,9-tetramethyl-[3R-(3.alpha.,5a.alpha.,9.alpha.,9a.alpha.)]-	21.462	50	2.07	222.37

Area %: area percent, which corresponds to the proportion of each component in the fraction; Qual %: quality of fragment ion peak matching between the unknown component and suggested compounds from NIST14.L; RT: retention time in min; MM: molecular mass. The “a” in superscript represents suggested compounds from NIST14.L which have Qual % less than 50%

## Discussion

The epicarp of tropical fruits has been reported to contain numerous phytochemicals which have been harnessed for human use in the pharmaceutical industries and in traditional medicine [24, 25]. Ajayi et al. [22] reported the potential protein targets of drug-like compounds from fractions of ethanol extract of the epicarp of *C. rostrata* and submitted that the epicarp contains phytochemicals with potential against pain and inflammatory diseases, diabetes and cancer. This study investigated the anti-proliferative and cytotoxic potential of the crude ethanol extract from the epicarp (1A) and ethanol extract of the leaf of *C. rostrata* (2A) against cancer cell lines: HeLa and MRC5-SV2.

1A showed significant cytotoxicity against both cell lines, but the effect was not time-dependent against HeLa or MRC5-SV2 cells. The cytotoxicity of the extract against HeLa was highest at the 50 μg/mL treatment. While the reason for this pattern was not entirely clear, it could be that at higher concentrations of 1A the perturbation of metabolic pathways in HeLa cells induced mechanisms that attenuated the effects, e.g., increased expression of transporters to pump out the phytochemicals [26]. The lack of additional cytotoxic effect at a longer time point could also be attributable to the nature of the cytotoxic components in the extract, e.g., their half-life in growth medium, their interactions with cellular components of dying cells or their interactions with normal waste products of cultured cell metabolism [27, 28]. The IC_50_ values for the cytotoxicity of 1A show that MRC5-SV2 cells are slightly more sensitive to the effect of the extract than HeLa cells.

The fractionation of 1A through solvent partitioning produced two fractions, 1AFK and 1AM, both of which showed lower cytotoxicity against both cell lines at 48 h. This suggests that, in 1A, the cytotoxic contents of the fractions had synergized with one another, resulting in lower IC_50_ values for the cytotoxicity of 1A against the two cell lines, following 48 h treatment, compared to the separate fractions producing IC_50_ values that were significantly higher against HeLa and MRC5-SV2 cells over the same exposure time. The effect of 1AFK on the cells involved the formation of pore-like damage on the cell membrane at 50 μg/mL and 100 μg/mL and it also induced significant oxidative stress and depolarization of the mitochondrial membrane (impairment of mitochondrial health). The findings suggest 1AM might act through other means than the generation of ROS, as it induced less than 2-fold increase in ROS. Data from the JC-1 staining assay also suggest that 1AM induces apoptosis (1AM was even more potent than 1AFK in reducing ∆Ψ_M_), which could be through means such as inhibition of intracellular antioxidant enzyme systems. It is also possible that, in some cases, lytic events were part of the cytotoxic response.

The leaf ethanol extract, 2A, showed significant cytotoxic activity against HeLa and MRC5-SV2 cells at 48 h, with IC_50_ values of 148.0 μg/mL ± 4.8 μg/mL and 165.0 μg/mL ± 8.8 μg/mL, respectively. Fractionation of the crude extract by solvent partitioning, VLC and column chromatography produced non-polar fractions (2AFU and 2AFH) with increased cytotoxicity, while the methanol fraction (2AFM), which had the highest proportion by mass in the crude, was less cytotoxic. A previous study on the leaf of *C. lepidota* (a close variety) by Oghenerobo and Falodun [29] reported high DPPH radical-scavenging activity of the chloroform and methanol extracts. The relatively non-cytotoxic effect of 2AFM is consistent with the findings of Oghenerobo and Falodun [29], and a high antioxidant activity may be the rationale for its low cytotoxicity. While the mechanism of action of fraction 2AFU only involved a mild increase in ROS levels, the effects of the cytotoxic sub-fractions (2AFH25, 2AFH40LF and 2AFH50) obtained from the 2AFH fraction involved several-fold increases in ROS levels and/or impairment of mitochondrial activity as demonstrated by reduced ∆Ψ_M_.

The GC-MS analysis of 2AFH50 identified saturated short- to medium-chain natural fatty acids as the major components; the proportion of palmitic acid (hexadecanoic acid) in 2AFH50 was 52%. Free fatty acids have been reported to disrupt the membrane architecture of human erythrocytes, affecting membrane fluidity and membrane protein functions [30]. A high concentration of palmitic acid has been reported to affect the membrane permeability of Caco-2 cells without inducing cytotoxicity or oxidative stress [31]. Increased incorporation of medium-chain fatty acids present in the extract into the membranes of HeLa and MRC5-SV2 cells might have sensitized the cells to the cytotoxic effects of other components in the extract. Palmitic acid was found to increase the production of nitric oxide in primary cultured rat skeletal muscle cells, and nitric oxide contributes to oxidative stress in many cells, including neurons [32, 33]. Though the effect of free palmitic acid on the viability of HeLa and MRC5-SV2 cells has not been investigated, literature reports support the argument that palmitic acid might have contributed to the cytotoxicity of 2AFH50 against HeLa and MRC5-SV2 cells.

In conclusion, cancer is a major public health challenge that necessitates the development of more efficacious and safer chemotherapeutic drugs. This work identifies *C. rostrata* as a potential source of new anti-cancer agents and further highlights the need to extensively screen more plants for bioactive principles that might represent novel scaffolds and deliver new chemical leads for drug discovery and development. Such endeavours can also produce fingerprints for the complex extracts and fractions which can be useful for quality control and authentication purposes, thus helping to achieve the standardisation necessary for the integration of traditional medicine into mainstream healthcare.

## Abbreviations

∆Ψ_M_: mitochondrial membrane potential
DCFDA: 2ʼ,7ʼ-dichlorofluorescein diacetate
DMSO: dimethyl sulfoxide
GC-MS: gas chromatography-mass spectrometry
JC-1: 5,5ʼ,6,6ʼ-tetrachloro-1,1ʼ,3,3ʼ-tetraethyl-imidacarbocyanine iodide
MTT: 3-(4,5-dimethylthiazol-2-yl)-2,5-diphenyltetrazolium bromide
NIST: National Institute of Standards and Technology
PBS: phosphate-buffered saline
ROS: reactive oxygen species
TBHP: tert-butyl hydroperoxide
VLC: vacuum liquid chromatography

## Declarations

### Availability of data and materials

Datasets are available from the authors upon reasonable request. Contact tobitune@yahoo.com or A.A.Fatokun@ljmu.ac.uk.

### Copyright

© The Author(s) 2023.
